# Motives for Participation and Amount of Physical Activity among Kelantan Chinese Adolescents

**DOI:** 10.21315/mjms2019.26.6.10

**Published:** 2019-12-30

**Authors:** Nurzulaikha Abdullah, Yee Cheng Kueh, Muhammad Hafiz Hanafi, Tony Morris, Garry Kuan

**Affiliations:** 1 Unit of Biostatistics and Research Methodology, School of Medical Sciences, Universiti Sains Malaysia, Kubang Kerian, Kelantan, Malaysia; 2 Department of Neuroscience, School of Medical Sciences, Universiti Sains Malaysia, Kubang Kerian, Kelantan, Malaysia; 3 Institute of Sport, Exercise and Active Living, College of Sport and Exercise Science, Victoria University, Melbourne, Australia; 4 Exercise and Sports Science Programme, School of Health Sciences, Universiti Sains Malaysia, Kubang Kerian, Kelantan, Malaysia; 5 Department of Life Sciences, Brunel University London, United Kingdom

**Keywords:** physical activity, motives, secondary students, exercise

## Abstract

**Background:**

Engaging in regular physical activity (PA) has become a worldwide issue for the prevention of numerous chronic diseases; therefore, is important to increase students’ desires to engage in PA by triggering their motivation. The purpose of this study was to examine the relationships between the motives for participating in PA and the amount of PA that secondary Chinese school students in Kelantan undertake.

**Methods:**

The participants consisted of 304 Chinese secondary school students (males = 165, females = 139) with a mean age of 13.55 years old (SD = 0.57) who volunteered to complete three measures, consisting of a demographic information form, the physical activity and leisure motivation scale for youth-Chinese version (PALMS-Y-C) and the Godin leisure-time exercise questionnaire-Chinese version (GLTEQ-C).

**Results:**

There were significant positive correlations between all the seven PA participation motives with amount of exercise (Enjoyment: *r* = 0.16, *P* = 0.010; Mastery: *r* = 0.23, *P* < 0.001; Competition: *r* = 0.21, *P* = 0.001; Affiliation: *r* = 0.22, *P* < 0.001; Psychological condition: *r* = 0.26, *P* < 0.001; Appearance: *r* = 0.20, *P* = 0.001; Physical condition: *r* = 0.20, *P* = 0.001). There were also significant mean differences among sweating exercise frequency categories in all the seven areas of PA participation motives (Enjoyment: *P* = 0.003, Mastery: *P* < 0.001, Competition: *P* = 0.001, Affiliation: *P* = 0.001, Psychological condition: *P* = 0.038, Appearance = 0.002, Physical condition: *P* = 0.004).

**Conclusion:**

The present study provided insight into how to promote PA in Kelantan Chinese school-aged children by specifically targeting their motives. Interventions targeting these motives could increase the amount of PA among Kelantan Chinese youths.

## Introduction

“Physical activity plays a vital role in balancing the energy expenditure with the energy consumption” (1). Contrarily, a sedentary lifestyle or a lifestyle with less or no physical activity (PA) is a leading factor in several health problems, like heart disease, diabetes, stroke, cancer and premature death (2). According to the National Health and Morbidity Survey conducted by the Institute for Public Health, the cases of physical inactivity in Malaysia reached 43.7% for the adult population, and almost one in five Malaysian adults have diabetes (3). Similarly, Turkey also has an issue with physical inactivity, and it exhibits a high rate of cardiovascular morbidity and mortality, despite the fact that it is a developing country (4, 5). This is because of many of the people living in Turkey are not aware that regular PA provides greater health benefits (6). Thus, it is crucial to promote PA and build a harmonious environment in order to minimise health-related diseases (4, 5).

The motives for PA participation are some of the most important factors for increasing the amount of PA; however, the PA participation rate among adolescents continues to decline (7–10). Moreover, a reduction in the PA amount has been linked with an increase in age (11). Using the multilevel ecological approach, recent initiatives in China, called ‘Healthy China 2030’, successfully created an active living community that can improve the physical exercise and physical fitness levels in Chinese children and adolescents (12, 13). To promote PA, greater initiatives should be implemented to motivate and increase the active lifestyle trend among adolescents (14, 15).

PA is beneficial to an overall healthy lifestyle, both physically and mentally, especially for adolescents. According to Shindler (16), reward or penalty is the common extrinsic motivational strategy for motivating behavioural change. However, he claimed that rewards work in behavioural change environments only. The Centres for Disease Control and Prevention further explained that PA health messages that encourage a healthy lifestyle should be made known to the public in a positive manner, so that more people will be interested in participating in the programme and staying healthy through adulthood (17).

PA helps improve psychological health and it maintains health-related fitness (8, 18). With regard to the early life stages, those adolescents who participated in regular PA showed higher scores in health-related fitness. They were more resistant to stress, and they had a better health-related quality of life (9, 18). Unfortunately, there are several barriers that contribute to physical inactivity, such as a lack of motivation, time constraints due to academic commitments, fatigue and a lack of confidence (11). Therefore, more effort should be made to increase the PA motivation among different groups of people. Those individuals with higher PA motivation levels enjoy PA more, and they participate in PA for a longer duration of time when compared to individuals with lower PA motivation levels.

The present study aimed to evaluate the adolescent students in secondary Chinese school students in Kelantan in terms of the relationship of their motives for participating in PA and the amount of PA undertake. This study is important because it will help us to further understand how motivation impacts the adolescent positively or negatively with regard to PA, which may influence their overall health. Those people with greater motivation tend to live a healthier lifestyle than those without the motivation to participate in exercise and PA. Moreover, it has been suggested that engaging in a sedentary lifestyle is one of the major risk factors for many diseases, such as diabetes and stroke (2). Many researchers have reported that adolescences have low PA levels, and this has become a concern, as stated by Hashim et al. (19) and Weiss (20). Aarnio et al. (21) suggested that young people are often considered to be the healthiest individuals in a population; however, their study showed that this generation’s health is at risk because they do not engage in PA consistently. Therefore, PA should be encouraged for all people, especially the youth, because young people who enjoy exercise and PA tend to stay active throughout their lives (22).

## Methods

### Research Design

A cross-sectional research design was employed in this one-year study (January–December 2018). This study was conducted at the Sekolah Menengah Jenis Kebangsaan Chung Hwa, in Kota Bharu, Kelantan, Malaysia.

### Participants

All participants were Kelantan secondary Chinese school students. Participants all spoke, read and wrote in Mandarin. A total of 304 adolescents (males = 165, females = 139) with an average age of 13.55 years old [standard deviation (SD) = 0.57] from Sekolah Menengah Jenis Kebangsaan Chung Hwa, Kelantan volunteered to participate in this study.

### Sample Size

Sample size determination was done by using power and sample size programme. The parameters used in the sample size determination were alpha = 0.05, power = 0.8, standard deviation of PA participation motives = 0.65 (22), mean difference between groups = 1.5 (based on experts’ opinion). The estimated sample size per group was 85. There were three comparison groups (i.e., sweating exercise frequency categories tested in a one-way ANOVA analysis), thus a total sample size of 255 was estimated for this study. After adding expected drop-out rate of 20% on the total sample size, a total of 319 (255/1–0.2) participants were needed for this study. The total completed set of questionnaires in the present study was 304 which was above the minimal target sample size (*N* ≥ 255). Thus, the sample size of 304 was considered adequate for the present study.

### Measures

#### Demographic questionnaire

Each participant was asked to complete a short demographic form. The form included the participant’s gender, age, class and year, PA participation duration in the past seven days, PA types and sports experience.

#### Physical activity and leisure motivation scale for youth-Chinese version (PALMS-Y-C)

The PALMS-Y-C (23) consists of 28 items with seven subscales measuring the different types of PA participation motives. The PALMS-Y-C has been validated by Hu et al. (23) in Chinese ethnic population in China. The PALMS-Y-C was reviewed by two local bilingual experts (i.e., English and Mandarin) in psychometric evaluation and sport psychology. The PALMS-Y-C was found suitable to be used in Malaysia’s secondary Chinese school students. The subscales include Mastery, Enjoyment, Psychological condition, Physical condition, Appearance, Affiliation and Competition. Each subscale contains five items, all measured on a 5-point Likert scale ranging from 1 (strongly disagree) to 5 (strongly agree). Higher scores reflect greater PA motivation. The internal consistency of each subscale was satisfactory with a Cronbach’s alpha ranging from 0.82 to 0.93 (23). The PALMS-Y was only available in Mandarin version (PALMS-Y-C) during the data collection period, therefore a secondary Chinese school in Kota Bharu was selected as a study location for the present study (23).

#### Godin leisure-time exercise questionnaire-Chinese version (GLTEQ-C)

The GLTEQ-C (24) consists of four self-explanatory questions about the subjects’ usual leisure-time exercise habits. The English version of GLTEQ was translated into Mandarin version (GLTEQ-C) for the present study. The preliminary version of GLTEQ-C was reviewed and finalised by two experts in psychometric evaluation and sport psychology. The experts were bilingual speakers in English and Mandarin. The GLTEQ has been used widely by researchers to examine the weekly leisure activity level. The questions focus on the average frequency of strenuous exercise (G1), moderate exercise (G2) and mild exercise (G3), with an additional question (G4) about how often the respondents exercise ‘long enough to work up a sweat’. Sweating exercise frequency which is measured by G4 is the frequency of participants exercised long enough to work up a sweat or experience sweating. Sweating exercise frequency was measured in an ordinal level (i.e., Often, Sometimes, Never). The measure is reliable with a Cronbach’s alpha of 0.64. Using the Kappa index, the reliability was 0.94 for the strenuous activity score and 0.74 for the total leisure-time physical activities (LTPA) score (24). Amireault et al. (25) conducted a systematic review using the GLTEQ and they found good evidence of validity when using the intraclass coefficient, which ranged from 0.98 to 1.00. The formula used to calculate the total score was as follows: weekly leisure and exercise activity score = (9 × G1) + (5 × G2) + (3 × G3).

## Procedure

Ethics approval for this study was obtained from the Universiti Sains Malaysia Human Research Ethics Committee. The study was conducted in accordance with the guidelines of the International Declaration of Helsinki. A convenience sampling method was applied by selecting students at the secondary Chinese school in Kota Bharu who were available to fill in the questionnaires during the data collection period.

The participants were briefed about the study purpose before the data collection and they were allowed to ask any questions related to the study. Then, the participant information sheet was provided, and informed consent was obtained from the participants and their parents. Those students who volunteered to participate in the study then completed the demographic form, the PALMS-Y-C and GLTEQ-C. After the forms were completed, they were returned to the researcher. We distributed a total of 320 questionnaires to the students; 303 were returned and usable for further analysis, after excluding the questionnaires with incomplete answers.

## Data Analysis

The statistical analyses were performed using IBM SPSS Statistics for Windows, version 22.0 (IBM Corp., Armonk, NY, USA). The data were checked for missing information, normality and outliers. Descriptive statistics means and SDs were used to describe the numerical demographic variables, PA participation motive levels (based on the mean score) and health-related fitness levels among the participants. All numerical variables were found to be normally distributed or approximately close to normal distribution. The frequency and percentage were used to describe the categorical demographic variables. A Pearson’s correlation was used to determine the relationships between the PA participation motives and the weekly PA amount. Simple and multiple linear regression were used to identify the factors associated with the weekly PA amount. A one-way analysis of variance was used to compare the sweating exercise frequency with the PA participation motives. Post-hoc comparison of the mean differences between the sweating exercise frequency categories on the PA participation motives was done by using Tukey’s post-hoc test. Level of significance was set at 0.05.

## Results

In this study, the majority of the participants were males (54.3%) and the participants’ mean age was 13.55 years old (SD = 0.57). Most of the participants were involved in some form of sporting activity, such as badminton (*n* = 71, 23.3%), jogging (*n* = 51, 16.8%), swimming (*n* = 43, 14.2%), walking (*n* = 24, 7.9%), football (*n* = 17, 5.6%) and others (*n* = 98, 32.2%). The mean PA motive levels ranged from the lowest for psychological condition [3.13 (SD = 0.69)] to the highest for enjoyment [4.14 (SD = 0.57)]. The descriptive statistics for the demographic variables and motivation subscales are listed in Table 1.

The descriptive statistics results for the GLTEQ-C values are shown in Table 2.

Pearson’s correlations were used to determine the relationships between the PA participation motives and PA amount. The results showed that there were significant positive correlations between the amount of exercise with all seven motives of PA participation (Table 3). The strength of the correlation varies from little to fair correlation.

In Table 4, the results showed that there were significant association between the PA participation motives and the weekly PA amount in a simple linear regression. However, motive of appearance was the only significant factor associated with weekly PA amount after adjusted for gender in a multiple linear regression. For female, they had 11.21 unit lower in weekly PA amount compared to their male counterparts. A unit increased in PA participation motive in terms of appearance, the weekly PA amount would increased by 8.09 unit.

In Table 5, the results showed that there were also significant differences in the PA participation motives based on the PA sweating exercise frequency levels (or categories) as measured by question G4 [i.e., during a typical 7-day period (a week), in your leisure time, how often do you engage in any regular activity long enough to work up a sweat]. There were significant differences between sweating exercise frequency categories (i.e., Often, Sometimes and Never) on each PA participation motives: enjoyment (*P* = 0.003), Mastery (*P* < 0.001), Competition (*P* = 0.001), Affiliation (*P* < 0.001), Appearance (*P* = 0.002), Physical condition (*P* = 0.004) and Psychological condition (*P* = 0.038). Those who reported to have sweating exercise frequency with category ‘Often’ were generally higher in PA participation motives than those reported with ‘Never’ or ‘Sometimes’ (i.e., Mastery, Competition, Affiliation, Appearance, Physical condition and Psychological condition).

## Discussion

The PA participation motives play crucial roles in increasing the PA amount among school-going adolescents. A sufficient amount of PA is needed for maintaining a healthy lifestyle, good physical condition, better mental health, preventing sedentary lifestyle diseases and prolonging life expectancy (26, 27). Molanourozi et al. (28) reported that the PA motives are related to the PA amount. Additionally, Huotari et al. (29) emphasised the fact that greater PA participation motivation could lead to a greater PA amount. The efforts to promote PA among adolescents should begin by improving the PA participation motives among adolescents (28). The education and interactive exercise programmes at school should include the PA motivation elements, which can indirectly promote healthy and active lifestyles among adolescent students.

The mean PA participation motive levels in the present study varied from 3.13 to 4.14, which are similar to those from the study by Zach et al. (30), who reported that the PA participation motive levels ranged from 2.65 to 4.45. The mean PA participation motives in the present study were quite similar to those in the study conducted by Molanourozi et al. (28), who reported that the means of the PALMS subscales ranged from 3.66 (SD = 0.91, Competition) to 4.19 (SD = 0.61, Enjoyment). Overall, the highest motive level was seen in enjoyment, which is similar to the results reported by Lerner et al. (31). Based on interviews, Lerner et al. found that fun and enjoyment showed the highest correlations with PA (31). In conjunction with fun and enjoyment, it has been found that young people can develop traits, values and beliefs (socialisation) through engaging in PA with their peers (31). Thus, those individuals with higher PA participation motive levels were more likely to have a higher health-related fitness level.

The Pearson correlation results indicated that there was a significant positive correlation between amount of exercise with PA participation motives: enjoyment, mastery, competition, affiliation, appearance, physical condition, and psychological condition. This indicated that those who had higher level of PA participation motives had higher amount of exercise per week. There were also significant differences in the PA participation motives based on the sweating exercise frequency categories as measured by the question in the GLTEQ-C. The results showed that there were different PA participation motive levels based on the sweating exercise frequency type categories (i.e., Often, Sometimes and Never). In general, it indicated that those individuals who reported sweating exercises frequency as ‘Often’ exhibited the highest PA participation motivation levels.

By improving students’ PA participation motives, their PA duration may be enhanced in daily life, indirectly enhancing their health-related fitness, which could promote active and healthy lifestyles among adolescents. The mean PA amount from the present study was 39.49 (METs/week), which was lower than the results reported by DeLong, which were 51.5 METs for 150 min per week (32). However, this may due to the different study population used in DeLong’s study. The study population consisted of college students, and all of them had athletic club memberships/access at their college.

Malina suggested that individuals who are active during their childhood may remain active through adulthood, which is a good healthy lifestyle habit (33). Many researchers have reported the benefits of engaging to PA (34) and different PA types and amounts are required for different health outcomes (1). This information could help make people more aware of the usefulness of exercise and help them to reduce their sedentary lifestyles. Their commitment could also encourage a better, healthier lifestyle in the younger generation.

Secondary school is the ‘right’ phase, between primary and tertiary school, to initiate a preparation by providing a social and academic environment to suit the transition from early childhood to adulthood; however, the transition to high school is particularly difficult for students who are academically or socially deficient (35). Student-school bonding via participation in extracurricular activities can help to reduce student behaviour problems (35). Therefore, it is important to encourage adolescents to engage in PA to promote healthy lifestyles in the future.

## Limitation

The present study did have some limitations. First, the sample size focused on a specific population: Chinese secondary school students in Kota Bharu, Kelantan. Thus, the results can only be generalised to a similar population. For future research, a much larger sample size is needed so that the results can be generalised to a wider population by including other ethnicities in Malaysia. Second, information bias may have occurred during the data collection. The participants may have provided dishonest answers when they were filling out the questionnaire. Moreover, they may have responded to the questionnaire in such a way that the answers were favourable to the expectations of society.

## Conclusion

In conclusion, the PA participation motives were significantly related to the exercise amount as measured by the GLTEQ-C and they varied based on the sweating exercise frequency category. All the PA participation motives were related to the exercise amount. Overall, it is important to instil a culture of PA engagement by triggering motivation, which could improve the exercise amount. This improvement could indirectly encourage healthy lifestyles among adolescent students.

## Figures and Tables

**Table 1:** Participants’ demographic data and physical activity motivation scale values

Variables	Mean (SD)	Frequency (%)
Age	13.55 (0.57)	
Gender		
Male		165 (54.3)
Female		139 (45.7)
Physical activity motivations		
Enjoyment	4.14 (0.57)	
Mastery	3.54 (0.64)	
Competition	3.65 (0.61)	
Affiliation	3.87 (0.62)	
Psychological	3.13 (0.69)	
Physical	3.52 (0.68)	
Appearance	3.55 (0.641)	

**Table 2:** Descriptive statistics for the GLTEQ-C

Variables	Mean (SD)	*n* (%)
G1 (Strenuous)	2.34 (1.94)	
G2 (Moderate)	2.42 (1.31)	
G3 (Light)	2.11 (1.86)	
Total leisure and exercise activity score [metabolic equivalent of task (MET) × hours/week]	39.49 (21.30)	
G4 (Sweating exercise frequency levels)		
Often		89 (30.1)
Sometimes		191 (64.5)
Never		16 (5.4)

G = Godin question

**Table 3:** Correlations between the PA participation motives and the weekly PA amount

PA participation motives	Correlation with the PA amount, *r*	*P*-value
ENJ	0.16	0.010
MAS	0.23	< 0.001
COM	0.21	0.001
AFF	0.22	< 0.001
PSY	0.26	< 0.001
APP	0.20	0.001
PHY	0.20	0.001

Notes: ENJ = Enjoyment, MAS = Mastery, COM = Competition, AFF = Affiliation, PSY = Psychological condition, APP = Appearance, PHY = Physical condition, *r* = Pearson’s correlation coefficient, *P*-value < 0.05 shows a significant correlation

**Table 4:** Factors associated with weekly PA amount

Variables	Simple linear regression		Multiple linear regression	
	B (95% CI)	*P*-value	B (95% CI)	*P*-value
ENJ	5.66 (1.37, 9.96)	0.010		
MAS	7.68 (3.82, 11.53)	< 0.001		
COM	7.33 (3.18, 11.48)	0.001		
AFF	7.47 (3.51, 11.44)	< 0.001		
APP	8.58 (4.78, 12.38)	< 0.001	8.09 (4.31, 11.86)	< 0.001
PHY	6.10 (2.39, 9.80)	0.001		
PSY	6.03 (2.44, 9.63)	0.001		
Gender		< 0.001		< 0.001
Male	0		0	
Female	−11.50 (−16.36, −6.64)		−11.21 (−16.13, −6.29)	

**Table 5:** Frequency evaluation of sweating exercise based on PA participation motives

Physical activity motives	Sweating exercise frequency categories	Mean (SD)	*F*-stat (df)	*P*-value^a^
ENJ	Never	3.78 (0.50)	6.01 (2, 290)	0.003
	Sometimes	4.11 (0.59)		
	Often	4.28 (0.60)		
MAS	Never	3.80 (0.70)	9.89 (2, 290)	< 0.001
	Sometimes	3.46 (0.61)		
	Often	3.30 (0.61)		
COM	Never	3.85 (0.65)	7.43 (2, 288)	0.001
	Sometimes	3.57 (0.60)		
	Often	3.42 (0.48)		
AFF	Never	4.06 (0.72)	7.73 (2, 291)	0.001
	Sometimes	3.81 (0.58)		
	Often	3.50 (0.59)		
APP	Never	3.77 (0.73)	6.30 (2, 285)	0.002
	Sometimes	3.48 (0.61)		
	Often	3.40 (0.69)		
PHY	Never	3.69 (0.75)	5.74 (2, 287)	0.004
	Sometimes	3.50 (0.64)		
	Often	3.10 (0.62)		
PSY	Never	3.29 (0.74)	3.31 (2, 290)	0.038
	Sometimes	3.07 (0.65)		
	Often	3.14 (0.61)		

Notes:

a One-way analysis of variance applied, Post-hoc comparison result: ENJ (Often versus Never: *P* = 0.007), MAS (Often versus Never: *P* = 0.016; Often versus Sometimes: *P* < 0.001), COM (Often versus Never: *P* = 0.042; Often versus Sometimes: *P* = 0.002), AFF (Often versus Never: *P* = 0.005; Often versus Sometimes: *P* = 0.009), APP (Often versus Sometimes: *P* = 0.003), PHY (Often versus Never: *P* = 0.008) and PSY (Often versus Sometimes: *P* = 0.038)
